# Examining sex as a potential moderator of metacognitive training for psychosis efficacy on cognitive insight and jumping to conclusions bias: Evidence from a large-scale harmonized database

**DOI:** 10.1016/j.scog.2026.100440

**Published:** 2026-04-27

**Authors:** Adrien Goncalves, Maria Lamarca, Steffen Moritz, Łukasz Gawęda, Vanessa Acuña, Caroline König, Fabrice Berna, Susana Ochoa

**Affiliations:** a INSERM, UMR_S 1329: Strasbourg Translational Neuroscience & Psychiatry, Strasbourg, France; b University of Strasbourg, École doctorale des sciences de la vie et de la santé: ED 414, Strasbourg, France; c Parc Sanitari Sant Joan de Déu, Sant Boi de Llobregat, 08830, Barcelona, Spain; d Grup MERITT, Institut de Recerca Sant Joan de Déu, Esplugues de Llobregat, 08950, Barcelona, Spain; e Consorcio de Investigación Biomédica en Red de Salud Mental (CIBERSAM), Instituto de Salud Carlos III, Madrid, Spain; f Departament de Psicologia Clínica i de la Salut, Facultat de Psicologia, Universitat Autònoma de Barcelona, Bellaterra, Cerdanyola del Vallès, 08193, Barcelona, Spain; g Department of Psychiatry and Psychotherapy, University Medical Center Hamburg, Hamburg, Germany; h Experimental Psychopathology Lab, Institute of Psychology, Polish Academy of Sciences, Warsaw, Poland; i Departamento de Psiquiatría, Escuela de Medicina, Facultad de Medicina, Universidad de Valparaíso, Valparaíso, Chile; j Soft Computing Research Group at Intelligent Data Science and Artificial Intelligence Research Center, Universitat Politècnica de Catalunya, Barcelona, Spain; k University of Strasbourg, Faculty of medicine, Strasbourg, France; l Hôpitaux universitaires de Strasbourg, Strasbourg, France

**Keywords:** Metacognitive training, MCT, Psychosis, Sex differences, Insight, Jumping to conclusions

## Abstract

**Background:**

Whereas biological sex differences in psychosis are well-documented in terms of clinical presentation and illness course, their moderating role in the effectiveness of cognitive interventions remains unclear. Previous studies in first-episode psychosis (FEP) suggested sex-specific responses to Metacognitive Training for psychosis (MCT) on cognitive insight and jumping to conclusions (JTC) bias, but generalizability to broader clinical populations is unknown.

**Methods:**

This retrospective study analyzed harmonized individual participant data from 633 persons with schizophrenia spectrum disorders (SSD) who received MCT across 22 international studies. Treatment effects and potential sex moderation were analyzed using repeated-measures ANOVAs and mixed-effects logistic regression on cognitive insight (Beck Cognitive Insight Scale) and JTC bias (Cognitive Biases Questionnaire for Psychosis, JTC subscale; Beads tasks).

**Results:**

MCT produced significant improvements in cognitive insight and JTC bias, including reduced self-certainty (*F* = 22.899, *p* < .001), improved composite cognitive insight score (*F* = 11.787, *p* < .001), and decreased JTC bias on both continuous (*F* = 4.109, *p* = .044) and dichotomous measures (OR = 0.592, 95% CI: 0.356–0.984). However, no significant time × sex interactions were observed for any outcome, indicating equivalent treatment benefits across sexes.

**Conclusions:**

Contrary to previous FEP-specific findings, sex does not moderate MCT efficacy in heterogeneous clinical samples including established schizophrenia. These results may support MCT implementation without sex-specific adaptations in routine clinical practice.

## 1 Introduction

Schizophrenia spectrum disorder (SSD) is a severe mental health condition characterized by positive symptoms, negative symptoms, and disorganized thinking (Jauhar et al., 2022). Cognitive theories suggest that cognitive biases contribute to the emergence and maintenance of psychotic symptoms, such as delusions and hallucinations (A. T. Beck and Haigh, 2014; Gawęda et al., 2024). Among these, the jumping to conclusions (JTC) bias, defined as a tendency to make decisions based on limited information (Ross et al., 2015), has been associated with clinical outcomes and recovery trajectories in SSD (Catalan et al., 2022; Hayashi et al., 2022; Mervis et al., 2022; Samson et al., 2024). Another key bias is cognitive insight, which refers to the capacity to evaluate the limitations of one's own thinking processes and beliefs (Van Camp et al., 2017) and has also been linked to clinical outcomes (Canal-Rivero et al., 2022; Hazan et al., 2025).

Therapies targeting such cognitive processes have become widely developed and more accessible (Moritz and Lysaker, 2018; Sitko et al., 2020; Moritz et al., 2025; Violeau et al., 2025). Among them, Metacognitive Training (MCT) for psychosis, developed by Moritz and Woodward (2007), is a psychological intervention derived from cognitive behavioral therapy (CBT), including a psychoeducational approach. This low-intensity metacognitive program is specifically designed to increase awareness of one's cognitive biases, including JTC. Rather than directly addressing delusions or perceptual experiences, MCT uses neutral and engaging tasks to explore underlying cognitive mechanisms. This approach makes it suitable for individuals with low levels of insight or motivation, who often respond positively with high satisfaction (Acuña et al., 2024). Various MCT versions are available (http://uke.de/mct), and the intervention has demonstrated efficacy across various clinical settings, including improvements in psychotic symptoms (Goncalves et al., 2025; Meinhart et al., 2025), cognitive insight (Lopez-Morinigo et al., 2020; Sauvé et al., 2020; Houdebine et al., 2025) and reductions in cognitive biases (Penney et al., 2022).

While biological sex differences in psychosis are relatively well-documented in terms of clinical presentation, including age of onset, symptom severity, illness trajectory and social functioning (Seeman, 2021; Usall and Ochoa, 2024; Moniem and Kafetzopoulos, 2025), their potential moderating role in response to psychological interventions remains largely unexplored. These differences are partly attributed to neurobiological mechanisms, including the neuroprotective role of estrogens, which regulate dopaminergic activity in mesocortical and mesolimbic pathways as well as dopamine availability in prefrontal circuits (Brand et al., 2021). Given that JTC bias has been theorized to involve dopaminergic dysregulation in delusion formation (albeit without conclusive empirical support to date) (Broyd et al., 2017), and that estrogen deficiency is associated with increased psychotic vulnerability (Brand et al., 2021), sex-related neurobiological differences may constitute a relevant context for cognitive variability in SSD. Although meta-analyses report small to medium effect sizes for most cognitive sex differences in the general population (Kheloui et al., 2023), the neurobiological alterations characteristic of SSD, may create conditions in which such differences become more clinically relevant than in non-clinical samples (Fond et al., 2018).

In this context, cognitive insight and JTC bias may differ by biological sex, highlighting the importance of assessing these constructs using appropriate measures (Espinosa et al., 2024; Díaz-Cutraro et al., 2025). Cognitive insight is most commonly assessed using the Beck Cognitive Insight Scale (BCIS), which includes two dimensions: self-reflectiveness (recognition of one's fallibility and willingness to correct judgments) and self-certainty (overconfidence in one's judgments) (Beck, 2004). JTC bias is typically assessed using probabilistic reasoning tasks, in which participants must decide from which of two sources a sequence of stimuli is drawn based on limited information (Ross et al., 2015; Dudley et al., 2016), or via self-report questionnaires such as the CBQp (Peters et al., 2014).

Considering broader sex differences in clinical presentation and functioning, treatment needs may also differ between male and female with psychosis (Ochoa et al., 2001; Comacchio et al., 2019). Some studies suggest differential responses to psychological treatment, particularly in engagement, insight and symptoms outcomes (Brabban et al., 2009; Hansen et al., 2012; Hardy et al., 2022). Regarding MCT specifically, early studies in first-episode psychosis (FEP) reported that reductions in self-certainty were greater in females, whereas reductions in JTC bias were observed in males but not in females (González-Blanch et al., 2021; Salas-Sender et al., 2020). A recent meta-analysis also found greater improvements in self-reflectiveness in females, although this effect did not remain significant in sensitivity analyses restricted to randomized controlled trials, underscoring the need for adequately powered investigation (Gelner et al., 2025). Taken together, these findings suggest that biological sex is among the most consistently investigated candidate moderators of MCT response, although the evidence remains preliminary, with meta-regression analyses also reporting null findings (Penney et al., 2022). In contrast, other demographic variables such as age, duration of illness, and medication dose have not shown consistent effects on MCT outcomes (Penney et al., 2022; Gelner et al., 2025), and gender as a social construct (Clayton and Tannenbaum, 2016) has not been examined to date, as existing studies have only reported data based on biological sex assigned at birth. One exception is years of education, which has been identified as a significant moderator of MCT effects on self-certainty in one meta-analysis (Gelner et al., 2025), though this finding requires replication. Accordingly, the present study focuses specifically on biological sex as the primary moderator of interest, given the existence of a priori directional hypotheses derived from FEP studies (Salas-Sender et al., 2020; González-Blanch et al., 2021) and because examining multiple moderators simultaneously would increase the risk of Type I error in the absence of strong a priori hypotheses (Barnett et al., 2022). Identifying whether sex modulates the effects of MCT is therefore of considerable importance for optimizing care, either by supporting standardized implementation or by justifying the development of adapted versions (König et al., 2025b; Lamarca et al., 2026), within a precision psychiatry framework (Scala et al., 2023).

To address these questions, the present study relies on a harmonized database from the European PERMEPSY project (www.permepsy.org). This individual participant data (IPD) approach provides several advantages: large sample size for detecting moderating effects, diverse clinical and geographical contexts strengthening external validity, and access to variables rarely available in individual studies (Riley et al., 2021). The aim is to explore whether cognitive recovery trajectories are sex-specific, using cognitive insight and JTC bias as primary outcomes. We hypothesize that improvements in self-certainty will be greater in females, whereas reductions in JTC bias will be greater in males.

## 2 Methods

### 2.1 Data source and participants

This study uses IPD from the PERMEPSY harmonized database (European ERAPERMED project: “Towards Personalized Medicine for Psychosis”). The harmonized database was designed to retain only variables identified as relevant to MCT outcome prediction, following a structured multi-step harmonization pipeline (König et al., 2025a). The dataset includes information from 698 individuals diagnosed with SSD who underwent MCT across 22 international studies (Supplementary Table S1), including pre- and post-treatment evaluations of: 1. clinical symptoms (Positive and Negative Syndrome Scale – PANSS; Kay et al., 1987); 2. cognitive insight (Beck Cognitive Insight Scale – BCIS; Beck, 2004); 3. JTC bias assessed through the Cognitive Biases Questionnaire for Psychosis JTC subscale - CBQp-JTC (Peters et al., 2014) and dichotomous draw-to-decisions (DTD) measures derived from Beads-type tasks (P. A. Garety et al., 1991). Additionally, the dataset includes sociodemographic variables such as age, sex, education, living situation, and employment status.

Inclusion criteria: (1) age ≥ 16 years; (2) diagnosis of SSD; (3) availability of at least one pre- and post-treatment cognitive outcome measure (BCIS or JTC). Exclusion criteria: severe organic brain dysfunction or learning disability. Of the 698 individuals in the PERMEPSY harmonized database, 633 met all inclusion criteria and were retained for analysis.

### 2.2 Data harmonization

The harmonization process followed a structured multi-step pipeline described in detail in König et al., (2025a), comprising: (1) selection of data sources; (2) identification of target variables of interest; (3) variable mapping across datasets using a common taxonomy; (4) application of transformation rules to bring variables into a common format; and (5) data cleaning. Python scripts in Jupyter Notebooks were used to automate and ensure reproducibility of steps 3–5. Sociodemographic variables were standardized using predefined categorical systems (e.g., employment coded 1–7, living status 1–4). Psychosocial indicators were systematically recalculated from item-level data when available, with range verification and replacement of erroneous entries with NA. To address variability across datasets, only pre- and post-treatment evaluations were retained, as these were the most consistently available timepoints across studies (intermediate and follow-up evaluations were excluded). Only MCT patients were integrated, as heterogeneity in control conditions precluded valid harmonization of control data.

Following this harmonization process, the variables retained for the present analyses were selected to operationalize the two primary constructs of interest (cognitive insight and JTC bias) alongside the primary moderator variable:

‐ Biological sex at birth (male/female), used as the primary moderator variable.
‐ BCIS comprises two subscales: Self-Reflectiveness (9 items, range 0–27) and Self-Certainty (6 items, range 0–18), for a total of 15 items rated on 4-point Likert scales (range 0–45). Higher Self-Reflectiveness or lower Self-Certainty indicates better cognitive insight. The composite BCIS (Self-Reflectiveness minus Self-Certainty, range − 18 to +27) reflects better insight with higher scores.
‐ CBQp consists of 30 items assessing five cognitive biases: JTC, intentionalising, catastrophising, emotional reasoning, and dichotomous thinking. Each item uses a 3-point Likert scale, with higher scores indicating stronger bias. Only the 6-item JTC subscale (CBQp-JTC, range 6–18) was used here.
‐ The DTD constitutes an independent dichotomous assessment of JTC bias, derived from probabilistic reasoning tasks (Fish Task and Beads Task). Participants decide from which source (lake/jar) a sequence is drawn. The cutoff of fewer than three draws as indicative of JTC bias was based on established scoring guidelines (P. Garety et al., 2013), and was coded as a binary variable (≤2 draws = 1; otherwise = 0).

### 2.3 Intervention

MCT for psychosis is a structured intervention with 8–10 modules addressing cognitive biases including JTC, attributional style, belief inflexibility, and overconfidence in errors. It helps individuals with psychosis reflect on thinking patterns and recognize cognitive distortions by adopting a reflective stance toward their cognition. It incorporates interactive exercises to promote metacognitive awareness, enabling participants to better understand the relationship between thought processes and symptoms.

In the PERMEPSY harmonized dataset, participants received different versions of MCT depending on study design and clinical context: the original 8-session group-based MCT, the new 10-session group-based MCT, the one-to-one individualized MCT+ format, or MCT-N (a variant tailored to negative symptoms, Swanson et al., 2022). Most studies included in this dataset have used the 8-module version of group MCT without the new COGITO App (https://clinical-neuropsychology.de/cogito-en/), which supports the learning objectives of the MCT program. Despite format differences, all versions share a unified therapeutic framework focused on enhancing metacognitive awareness regarding cognitive biases.

### 2.4 Data analysis

Statistical analyses were carried out to evaluate the impact of MCT on cognitive outcomes (cognitive insight and JTC bias), with attention to sex-related differences in treatment response. The primary strategy involved repeated-measures ANOVAs applied individually to each psychological variable. These analyses assessed the influence of Time (pre- vs. post-treatment), Sex, and the Time × Sex interaction on cognitive insight and JTC bias. Time served as the within-subjects factor while Sex was the between-subjects factor. Despite non-normal distributions (Shapiro-Wilk, all *p* < .001), we used ANOVAs given the large sample (*N* > 221) ensuring the analyses would remain robust. Levene's test revealed heterogeneity of variance for BCIS Self-Certainty pre-treatment (*F* = 5.759, *p* = .017); all other measures satisfied homogeneity assumptions (all *p* > .05).

For DTD measures (dichotomous format), we conducted mixed-effects logistic regression. Model assumptions were verified and validated. Collinearity analysis revealed acceptable VIF values for all predictors (Time: 1.64; Sex: 2.03; Time × Sex: 2.68), indicating no problematic multicollinearity. Diagnostic plots confirmed linearity and normality of random effects. The model converged successfully with no overdispersion (χ^2^/DF = 0.25).

All analyses were conducted in Jamovi, with effect sizes (*η*^*2*^) and two-tailed significance levels reported (*p* < .05). Post hoc power analyses were conducted using G*Power 3.1.9.6 (Faul et al., 2007) to verify adequate sample sizes for detecting meaningful effects in repeated measures ANOVAs, assuming an alpha level of 0.05 and a correlation of 0.5 between repeated measures.

Given previous findings that sex moderates MCT treatment effects in FEP on both insight and JTC bias, we conducted post-hoc exploratory analyses to examine Time × Sex interactions in our FEP subsample. Due to limited data, these analyses were restricted to BCIS measures (*n* = 49–50 depending on subscales) and could not be conducted for JTC bias measures. Post-hoc power analyses were conducted using G*Power 3.1.9.6 to assess statistical sensitivity of these FEP comparisons.

To assess the robustness of primary findings to potential confounding, sensitivity analyses were conducted by adding years of education as a covariate in all repeated-measures ANOVAs (ANCOVAs). Education was selected as the only demographic variable with empirical support as a moderator of MCT outcomes in the existing literature (Gelner et al., 2025). These analyses were restricted to participants with available education data and are reported in Supplementary Table S2.

Missing data patterns were examined by testing whether sex was associated with post-treatment data availability for each outcome measure, using chi-square tests of independence. Results are reported in Supplementary Table S3. No significant association between sex and data availability was found for any outcome, with negligible effect sizes. These results indicate that missingness was not differentially distributed across sexes, supporting the validity of moderation analyses. Consistent with the retrospective harmonization design and multi-site origin of the dataset, data were assumed to be missing at random (MAR; Riley et al., 2021). All analyses were conducted using complete case analysis.

## 3 Results

### 3.1 Sample characteristics

The final sample comprised 633 participants with SSD who completed at least one pre-treatment cognitive insight (either BCIS subscale) or JTC bias assessment (CBQp-JTC or DTD). Sample demographic and clinical characteristics are shown in Table 1.

**Table 1:** Sociodemographic and clinical characteristics of the retrospective sample.

	N	Mean (SD)	Missing	Min	Max
Age (in years)	629	36.6 (12.8)	4	16	69
Education (in years)	540	11.1 (3.17)	93	0	18
Illness duration (in years)	485	8.25 (10.7)	148	0	55
PANSS positive	506	15.1 (6.57)	127	7	39
PANSS negative	469	14.5 (6.24)	164	7	44
PANSS general	431	30.7 (9.62)	202	16	67
PANSS total	427	60.3 (19.3)	206	30	144


	N	% of Total	Missing
Sex			12
Male	378	60.9%	
Female	243	39.1%	
Employment			156
Inactive	164	34.4%	
Active	95	19.9%	
Retired	74	15.5%	
Temporal illness	62	13.0%	
Student	39	8.2%	
Disability	35	7.3%	
Others	8	1.7%	
Living situation			399
With family	180	76.9%	
Residence	24	10.3%	
Alone	22	9.4%	
Other	8	3.4%	
Diagnosis			12
Schizophrenia	340	56.2%	
Other SSD	128	20.6%	
Schizoaffective disorder	58	9.4%	
FEP	51	8.2%	
Delusional disorder	20	3.2%	
Brief psychotic disorder	9	1.4%	
Schizophreniform disorder	6	1.0%	

Note: PANSS = Positive and Negative Syndrome Scale; SSD = Schizophrenia Spectrum Disorder; FEP = First Episode Psychosis.

Repeated measures ANOVAs examined effects of time (pre vs. post), sex, and their interaction on continuous cognitive variables. Sample distributions for continuous measures are in Table 2. For dichotomous JTC bias (DTD < 3 vs. DTD > 2), mixed-effects logistic regression was conducted, and the descriptives of the sample's distribution are in Table 3.

**Table 2:** Descriptives of sample's cognitive insight and continuous JTC measures.

Measures	Sex	Pre			Post		
		N	Mean (SD)	Range	N	Mean (SD)	Range
BCIS Self-Reflection	Male	328	17.1 (4.77)	0–27	291	17.1 (5)	5–27
	Female	205	17.1 (4.6)	7–27	175	17.4 (4.68)	8–27
BCIS Self-Certainty	Male	332	10.7 (3.73)	2–18	291	10.2 (4.04)	0–18
	Female	202	10.7 (4.09)	0–18	175	10.1 (4)	2–18
BCIS Composite	Male	326	6.35 (4.82)	−12-21	289	6.88 (4.62)	−12 - 19
	Female	200	6.42 (4.9)	−6-23	174	7.27 (4.52)	−3 - 21
CBQp-JTC	Male	137	11 (2.1)	6–16	128	10.7 (2.11)	6–15
	Female	84	11.1 (1.95)	7–15	77	10.6 (2.08)	6–14

Note: BCIS = Beck Cognitive Insight Scale self-reflection; CBQp-JTC = Cognitive Biases Questionnaire for Psychosis jumping to conclusion subscale.

**Table 3:** Descriptives of sample's dichotomous JTC bias.

	Pre			Post		
	n JTC (% of total)	n non-JTC (% of total)	Total (% each sex)	n JTC (% of total)	n non-JTC (% of total)	Total (% each sex)
Male	128 (32.5%)	111 (28.2%)	239 (60.67%)	100 (31.2%)	94 (29.3%)	194 (60.44%)
Female	90 (22.8%)	65 (20.2%)	155 (39.34%)	65 (20.2%)	62 (19.3%)	127 (39.56%)
Total (% of total pre)	218 (55.33%)	176 (44.67%)	394 (100%)	165 (51.40%)	156 (48.60%)	321 (100%)

Note: JTC = Jumping To Conclusion.

### 3.2 Treatment effects

#### 3.2.1 Cognitive insight (BCIS)

Analysis on the composite BCIS revealed a significant main effect of time (*F* = 11.787, *p* < .001, *η*^*2*^_*p*_ = .026), indicating improved cognitive insight due to treatment. No significant sex effect (*F* = 0.445, *p* = .505, *η*^*2*^_*p*_ = .001) or time × sex interaction (*F* = 0.642, *p* = .423, *η*^*2*^_*p*_ = .001) emerged, suggesting equivalent treatment benefits across sexes.

Analysis on the self-reflection subscale revealed no significant main effect of time (*F* = 0.614, *p* = .434, *η*^*2*^_*p*_ = .001), indicating participants did not show increased inclination to question their own beliefs following treatment. No significant sex effect (*F* = 0.360, *p* = .549, *η*^*2*^_*p*_ = .0008) nor time × sex interaction (*F* = 0.407, *p* = .524, *η*^*2*^_*p*_ = .0009) emerged, suggesting that sex did not moderate treatment response on self-reflectiveness.

Analysis on the self-certainty subscale revealed a significant main effect of time (*F* = 22.899, *p* < .001, *η*^*2*^_*p*_ = .048), indicating treatment reduced participants' self-certainty and overconfidence. No significant sex effect (*F* = 0.046, *p* = .830, *η*^*2*^_*p*_ = .0001) nor time × sex interaction (*F* = 0.309, *p* = .579, *η*^*2*^_*p*_ = .0007) was observed, indicating that male and female participants experienced similar reductions in excessive certainty about their beliefs.

#### 3.2.2 JTC bias (continuous measures)

Analysis on CBQp-JTC showed a significant main effect of time (*F* = 4.109, *p* = .044, *η*^*2*^_*p*_ = .021), indicating reduced JTC bias following treatment. No significant sex effect (*F* = 0.0192, *p* = .890, *η*^*2*^_*p*_ = .0001) nor time × sex interaction (*F* = 0.279, *p* = .598, η^*2*^_*p*_ = .001) was detected, indicating that both male and female participants experienced similar reductions in JTC bias over the course of treatment.

#### 3.2.3 JTC bias (dichotomous measure)

Mixed-effects logistic regression on Beads-type tasks showed a significant time effect (χ^2^ = 4.08, *p* = .043), demonstrating reduced probability of JTC bias post-treatment (OR = 0.592, 95% CI: 0.356–0.984) with a 40.8% reduction in the odds of displaying the bias. Sex showed no significant main effect (χ^2^ = 0.405, *p* = .525), and the time × sex interaction was not significant (χ^2^ = 1.103, *p* = .294), indicating that treatment efficacy did not vary significantly between males and females.

### 3.3 Post hoc analysis

#### 3.3.1 Power analysis

Post hoc power analysis assessed study sensitivity given the final sample. For primary outcome, achieved power for detecting time effects ranged from 95% to 97% for BCIS composite and self-certainty subscales (*n* = 526–534), and 64% for CBQp-JTC (*n* = 221). Critically, all analyses demonstrated excellent power (≥ 98.8%) to detect moderate sex × time interactions (*f* = 0.143), providing compelling evidence that the absence of significant sex differences in treatment response was not due to insufficient statistical power. Power analysis was not conducted for the mixed-effects logistic regression on dichotomous JTC, as power calculation methods for complex mixed models require simulation-based approaches beyond this post-hoc analysis scope.

#### 3.3.2 Exploratory analyses in first-episode psychosis

No significant Time × Sex interactions emerged for any BCIS measure: composite index (*F* = 0.002, *p* = .965, *η*^*2*^_*p*_ = .00004), self-reflection (*F* = 0.57, *p* = .452, *η*^*2*^_*p*_ = .012), and self-certainty (*F* = 0.49, *p* = .486, *η*^*2*^_*p*_ = .01). Post-hoc power analyses using the same effect size threshold as for the primary analyses (*η*^*2*^ = 0.02, *f* = 0.143) revealed severely limited statistical power: 5% for composite index, 14% for self-reflection and 17% for self-certainty. Even if moderate-sized Time × Sex interactions existed in the FEP population (*f* = 0.143), the analyses would have had only 50–51% power to detect them. Given this insufficient statistical sensitivity, these null findings cannot be interpreted as evidence for the absence of sex-specific treatment effects in FEP patients.

## 4 Discussion

This study aimed to examine whether biological sex moderates the effects of MCT for psychosis on cognitive insight and JTC bias. Analyses showed significant post-treatment improvements across several measures, with small effect sizes: reduced self-certainty, improved composite BCIS index, and decreased JTC bias on both CBQp and Beads tasks. However, contrary to our initial hypotheses, no significant time and sex interaction emerged, suggesting equivalent therapeutic benefits across sexes.

This study presents several methodological strengths that reinforce the validity of its conclusions. First, this study relies on a harmonized database from 22 international studies. This IPD approach complements prior aggregate data meta-analyses (Penney et al., 2022; Gelner et al., 2025), which reported discordant findings regarding sex-specific effects on cognitive insight, highlighting the need for analyses conducted at the participant level. The use of IPD enabled standardization of outcome definitions and variable coding across all included datasets (Veroniki et al., 2023), ensuring consistency in the measurement of cognitive insight and JTC bias. It also provided access to participant-level variables such as biological sex, which are rarely systematically reported in published aggregate data, thereby enabling moderation analyses at the individual level (Tudur Smith et al., 2016). Second, our study used multiple complementary measures of JTC bias (CBQp and Beads tasks) and cognitive insight (BCIS subscales and composite index), allowing for triangulation of results that strengthens their robustness.

In relation to the existing literature, our findings do not fully align with pioneering FEP studies reporting sex-moderating effects (Salas-Sender et al., 2020; González-Blanch et al., 2021). Post-hoc exploratory analyses in the FEP subsample showed no significant Time × Sex interactions for any BCIS measure, but were severely underpowered and thus inconclusive. Moreover, the divergence between our results and previous FEP-specific studies may reflect fundamental population differences, particularly in illness stage. Our sample was substantially heterogeneous (56.2% schizophrenia, 8.2% FEP, mean illness duration 8.25 years), in contrast to the exclusively early-phase samples included in prior studies. Notably, the age of onset of SSD differs significantly between sexes. Males typically show peak onset in their early twenties, while females present a first peak in their late twenties, and a second one around menopausal age (Riecher-Rössler et al., 2018; Häfner, 2019; Selvendra et al., 2022). Females with later illness onset show fewer premorbid impairments than early-onset females or males (Allen et al., 2013; Dama et al., 2019; Ayesa-Arriola et al., 2020; Selvendra et al., 2022). Some studies even suggest poorer clinical outcomes in postmenopausal females compared to males and younger female counterparts, with increased relapse risks in older females (Sommer et al., 2023). Overall, longitudinal studies indicate outcome differences narrow or reverse over decades (Zhao et al., 2022), with sex differences most pronounced early and converging in late-life schizophrenia (Freeman and Lee, 2022; Moniem and Kafetzopoulos, 2025). Our findings in a predominantly chronic and heterogeneous sample are consistent with this developmental perspective, suggesting that sex-specific effects may diminish as illness progresses. While adequately powered studies are needed to determine whether sex moderates MCT efficacy in early psychosis, our results indicate that such effects, if present, may not persist into chronic SSD. These results may support standardized MCT application across sexes, particularly in established SSD, where most clinical care is delivered.

Despite its strengths, this study has several limitations. First, females are underrepresented in our database, which has unfortunately been historically the case in clinical trials across all health disciplines (Hodes and Kropp, 2023). Second, the retrospective harmonization approach, while providing a large sample, inevitably entails a loss of nuances specific to the original studies and substantial variability in intervention protocols (Riley et al., 2021). Participants received different versions of the program (standard MCT, MCT+, or MCT-N), with important variations in session numbers. Moreover, heterogeneity across studies in recruitment settings, baseline illness severity, cultural contexts, and concurrent treatments may have introduced unmeasured confounders that could mask true effects or create spurious associations. Third, not all our conclusions have the same statistical power, due to missing data on certain tests. For example, only 221 participants had complete CBQp data, considerably reducing the capacity to detect modest interactions. Additionally, missing variables across studies precluded reliable subgroup analyses stratified by illness stage. Fourth, the violations of normality assumptions observed across all measures (Shapiro-Wilk tests, all *p* < .001), although theoretically compensated by large sample size, call for some caution in interpreting results. Similarly, Levene's test indicated heterogeneity of variance for the BCIS Self-Certainty pre-treatment measure (*F* = 5.759, *p* = .017), suggesting findings related to this specific measure should be interpreted with appropriate caution. Fifth, the effect sizes observed for the main time effects, while statistically significant, are remarkably small (*η*^*2*^_*p*_ = .021–0.048), raising questions about their clinical significance (Carey et al., 2023). These results are consistent with the highest-level evidence conclusions regarding MCT's efficacy (Goncalves et al., 2025; Meinhart et al., 2025), which primarily find benefits on positive symptoms, particularly delusions. It is interesting to note that a program directly targeting cognitive aspects appears to achieve better results on clinical symptoms than on cognitive processes themselves, whereas these two domains appear strongly correlated (Gawęda et al., 2024). These observations could reflect a lack of sensitivity of cognitive processes instruments used, which may not fully capture real-world functioning. More generally, there is a lack of studies focusing on identifying the mechanisms behind CBT treatment effects (Quigley et al., 2019), highlighting the need for developing solutions to better understand the cognitive mechanisms through which metacognitive therapy impacts psychotic symptoms (Sheffield et al., 2024).

These findings open several promising research avenues. First, prospective studies specifically designed to investigate sex differences across illness stages would be valuable, employing stratified sampling to ensure adequate representation of both sexes at different phases of the disorder. Second, future research should explore the neurobiological and psychological mechanisms underlying cognitive processes according to sex. Functional neuroimaging studies could examine whether males and females present different patterns of brain activation during metacognitive tasks, even in the absence of manifest behavioral differences. Third, a study based on MCT+ has revealed that participants with jumping-to-conclusions bias and low self-esteem show particularly strong improvements in delusional severity and/or overall positive symptoms, while cognitive insight at baseline did not moderate the treatment efficacy relative to an active control group (Leanza et al., 2020). These findings suggest that the effectiveness of MCT may vary not only by sex differences but also by the interaction of sex with other clinical characteristics (Ferrer-Quintero et al., 2022). Future prospective studies with stratified sampling across illness stages and standardized protocols are needed to definitively establish whether sex moderation of cognitive processes is stage-specific or emerges through interactions with other baseline characteristics that may differentiate treatment response. More sophisticated subgroup analyses could potentially reveal differentiated response profiles that our main analyses did not detect. Finally, while the present study focused exclusively on biological sex at birth, future research should also examine the role of gender as a distinct social construct in response to metacognitive interventions, as these two dimensions interact to shape cognitive and behavioral processes (Kheloui et al., 2023). Indeed, psychosis may disrupt distinct aspects of life for males and females, with gender-specific experiences of stigma, social expectations, and identity losses that could influence therapeutic engagement (Firmin et al., 2021). Social norms and lived experiences related to gender could influence how individuals engage in metacognitive reflection and in MCT therapeutic exercises, independently of their biological sex (Usall and Ochoa, 2024). An intersectional perspective, also integrating sociocultural, economic, and ethnic dimensions, would allow moving beyond the sex/gender dichotomy and identifying the multiple psychosocial factors that modulate the efficacy of psychological interventions in psychosis.

## 5 Conclusion

This study examined whether biological sex moderates the efficacy of MCT for psychosis on cognitive insight and jumping-to-conclusions bias. Using a large harmonized database, we found significant treatment effects across all outcome measures: MCT reduced self-certainty, improved composite cognitive insight scores, and decreased jumping-to-conclusions bias on both continuous and dichotomous measures. Critically, no significant sex × time interactions were observed across any outcome, indicating that treatment benefits were equivalent for males and females. These results support the implementation of MCT without sex-specific adaptations in routine clinical practice, simplifying its dissemination and ensuring equitable benefits to this evidence-based intervention regardless of patient sex. Further research is needed to assess whether sex acts as a moderator of the effectiveness of MCT on other outcomes.

## CRediT authorship contribution statement

**Adrien Goncalves:** Writing – review & editing, Writing – original draft, Validation, Methodology, Formal analysis. **Maria Lamarca:** Writing – review & editing, Writing – original draft, Visualization, Validation, Methodology, Conceptualization. **Steffen Moritz:** Writing – review & editing, Validation. **Łukasz Gawęda:** Writing – review & editing, Validation. **Vanessa Acuña:** Writing – review & editing, Validation. **Caroline König:** Writing – review & editing, Validation. **Fabrice Berna:** Writing – review & editing, Validation, Supervision, Methodology, Conceptualization. **Susana Ochoa:** Writing – review & editing, Writing – original draft, Validation, Supervision, Methodology, Conceptualization.

## Ethics statement

Ethical approval for the use of anonymized retrospective data was obtained from the institutional review boards of all participating centers. Approval was granted by the Research Ethics Committee of Fundació San Joan de Déu on 27/04/2023 by approval PIC-68-23, the ‘Lokale Psychologische Ethikkommission am Zentrum für Pyschosoziale Medizin’ of UKE on 29/03/2023 by approval LPEK-0603, the ‘Comitè Ético Científico del Servicio de Salud Valparaíso San Antonio’ by approval No 54/2023 on 27/09/2023, and the ‘Ethics Committee of the UPC’ on 11/12/2023 by approval 2023.13. Prior to the harmonization process, all data were fully anonymized to protect participant confidentiality. Each study contributing to the retrospective dataset had received ethical clearance from the relevant ethics committees, and informed consent was obtained in accordance with the applicable local regulations. Both the individual studies and the overarching project responsible for the harmonization adhered to the ethical standards set forth in the most recent version of the Declaration of Helsinki.

## Funding

This article was carried out under the PERMEPSY project. The PERMEPSY project was supported under the frame of ERA PerMed (ANR-22-PERM-0009-05) by: 10.13039/501100004587Instituto de Salud Carlos III (ISCIII), Spain; 10.13039/501100002347Federal Ministry of Education and Research (BMBF), Germany; 10.13039/501100001665Agence Nationale de la Recherche (ANR), France; 10.13039/501100005632National Centre for Research and Development (NCBR), Poland; Agencia Nacional de Investigación y Desarrollo (ANID), Chile. The funding agencies were not involved in the execution of this study or in the evaluation of the results.

## Declaration of competing interest

This study is part of the PERMEPSY project, which involves SM, the main developer of MCT for psychosis. To mitigate potential conflicts of interest, SM was not involved in any activities related to data extraction or analyses. Nonetheless, we acknowledge that the involvement of key collaborators cannot fully rule out risk of allegiance bias.
