# Supplementary material for: Examining sex as a potential moderator of metacognitive training for psychosis efficacy on cognitive insight and jumping to conclusions bias: Evidence from a large-scale harmonized database
Source: Schizophr Res Cogn. 2026 Apr 27;45:100440. doi: 10.1016/j.scog.2026.100440 (PMC13137009; doi:10.1016/j.scog.2026.100440)
Supplement: Supplementary file 1 — Supplementary tables [file mmc1.docx]

**Supplementary Table 1.** Characteristics of the 22 studies included in the PERMEPSY database.

| **Main author** | **DOI** | **Year of publication** | **Country** | **Center** | **Setting** | **Sex**  **(♂ - ♀)** | **Mean age**  [Range] | **Diagnosis** | **MCT Format (participants)** | **Controls (participants)** |
| --- | --- | --- | --- | --- | --- | --- | --- | --- | --- | --- |
| Acuña | Unpublished | - | Chile | - | Outpatients | 29 - 17 | 27  [18 - 46] | SCZ | MCT (46) | - |
| Balzan | 10.1093/schbul/sby152 | 2019 | Australia | Mono | Outpatients | 32 - 22 | 37  [19 - 52] | SSD | MCT+ (27) | CR (27) |
| Favrod | 10.1016/j.jtcc.2015.06.002 | 2015 | Switzerland | Multi  (2) | Outpatients | 34 - 18 | 37  [19 - 59] | SSD | MCT (26) | TAU (26) |
| Fujii | 10.11596/asiajot.17.45 | 2021 | Japan | Mono | - | 10 - 7 | 54  [39 - 64] | SSD | MCT (9) | OT (8) |
| Gawęda | Unpublished | - | Poland | - | In and outpatients | 23 - 25 | 51  [30 - 69] | NS | MCT (25) | Discussion (23) |
| Ishikawa | [10.1016/j.schres.2019.08.006](https://doi.org/10.1016/j.schres.2019.08.006) | 2020 | Japan | Multi  (6) | In and outpatients | 25 - 25 | 48  [24 - 61] | SSD | MCT (24) | TAU (26) |
| Kuokkanen | 10.1002/cbm.1905 | 2014 | Finland | Mono | Inpatients  *(Forensic)* | 10 - 0 | 42  [28 - 55] | SCZ | MCT (10) | - |
| Leanza | 10.1016/j.jbtep.2020.101547 | 2020 | Germany | Mono | In and outpatients | 51 - 41 | 36  [18 - 67] | SSD | MCT+ (46) | CR (46) |
| Lopez-Morinigo | 10.1038/s41537-022-00316-x | 2023 | Spain | Mono | Outpatients | 41 - 36 | 47  [21 - 64] | SSD | MCT (39) | PE (38) |
| Moritz 1 | 10.1017/S0033291710002618 | 2011 | Germany | Mono | Inpatients | 31 - 17 | 34  [18 - 65] | SCZ | MCT+ (24) | CR (24) |
| Moritz 2 | 10.1016/j.schres.2013.10.007 | 2013 | Germany | Multi  (2) | In and outpatients | 97 - 56 | 35  [18 - 64] | SSD | MCT (79) | CR (74) |
| Moritz 3 | 10.1016/j.brat.2010.11.010 | 2011 | Germany | Multi  (2) | Outpatients | 28 - 8 | 33  [22 - 55] | SSD | MCT (18) | TAU (18) |
| Ochoa 1 | 10.1017/S0033291716003421 | 2017 | Spain | Multi  (9) | - | 85 - 37 | 27  [16 - 47] | SSD | MCT (65) | PE (57) |
| Ochoa 2 | NA | 2020 | Spain | Multi  (10) | - | 44 - 25  (1 NS) | 29  [18 - 45] | SSD | MCT+ (36) | TAU (34) |
| Ochoa 3 | Unpublished | - | Spain | - | - | 28 - 22  (4 NS) | 45  [21 - 65] | SSD | MCT (54) | - |
| De Pinho | 10.1111/jan.14627 | 2020 | Portugal | Multi  (4) | In and outpatients | 30 - 22 | 51  [30 - 66] | SCZ | MCT (26) | TAU (26) |
| Simon-Exposito | 10.3390/ijerph16224541 | 2019 | Spain | Multi  (2) | Inpatients | 16 - 6 | 45  [26 - 62] | SSD | MCT (11) | TAU (11) |
| So | 10.3389/fpsyg.2015.00730 | 2015 | China | Mono | Outpatients | 24 - 20 | 33  [17 - 62] | SSD | MCT+ (23) | TAU (21) |
| Swanson | 10.1002/cpp.2692 | 2021 | Scotland | Mono | In and outpatients | 13 - 2 | 42  [27 - 67] | SSD | MCT-N (15) | - |
| Tanoue | Unpublished | - | Japan | - | Outpatients | 14 - 8 | 51  [30 - 68] | SCZ | MCT (22) | - |
| Ussorio | 10.1111/papt.12059 | 2016 | Italy | Mono | Outpatients  *(Early intervention)* | 30 - 13 | 22  [16 - 32] | SSD | MCT (43) | - |
| Yildiz | 10.29399/npa.23095 | 2019 | Turkey | Mono | Outpatients | 13 - 7 | 35  [21 - 56] | SSD | MCT (10) | PST (10) |

*Note. MCT = MetaCognitive Training (group); MCT+ = MetaCognitive Training (individual); MCT-N = MetaCognitive Training (group) - variant targeting negative symptoms; CR = Cognitive remediation; OT = Occupational therapy; PE = Psychoeducation; PST = Psychosocial Skills Training; TAU = Treatment as usual; SSD = Schizophrenia spectrum disorder; SCZ = Schizophrenia; NA = Not available.*

**Supplementary Table 2.** Sensitivity analyses: repeated-measures ANCOVAs with years of education as a covariate.

| **Outcome** | ***F***  Time | ***p***  Time | ***F***  Time×Sex | ***p***  Time×Sex | ***F***  Time×Education | ***p***  Time×Education |
| --- | --- | --- | --- | --- | --- | --- |
| BCIS Self-Reflection | 0.614 | .434 | 0.407 | .524 | — | — |
| + Education | 0.980 | .323 | 0.309 | .579 | 0.508 | .477 |
| BCIS Self-Certainty | 22.899 | <.001 | 0.309 | .579 | — | — |
| + Education | 2.874 | .091 | 0.003 | .957 | 0.231 | .631 |
| BCIS Composite | 11.787 | <.001 | 0.642 | .423 | — | — |
| + Education | 3.515 | .062 | 0.132 | .717 | 0.833 | .362 |
| CBQp-JTC | 4.109 | .044 | 0.279 | .598 | — | — |
| + Education | 0.546 | .461 | 0.002 | .961 | 1.117 | 0.292 |

Analyses were restricted to participants with available education data (n = 383 - 462 depending on the outcome). Education was entered as a continuous covariate. Participants with unknown sex (n = 12) were excluded from all analyses.

**Note:** *BCIS = Beck Cognitive Insight Scale; CBQp-JTC = Cognitive Biases Questionnaire for Psychosis jumping-to-conclusions subscale.*

**Supplementary Table 3.** Chi-square tests of independence between sex and data availability of post-treatment outcome data.

| **Outcome** | **Males**  Data available *n* (%) | **Females**  Data available *n* (%) | **χ²** | ***p*** | **Cramér's V** |
| --- | --- | --- | --- | --- | --- |
| BCIS Self-Reflection | 291 (77.0%) | 175 (72.0%) | 2.28 | .131 | .061 |
| BCIS Self-Certainty | 291 (77.0%) | 175 (72.0%) | 2.28 | .131 | .061 |
| BCIS Composite | 289 (76.5%) | 174 (71.6%) | 2.15 | .143 | .059 |
| CBQp-JTC | 128 (33.9%) | 77 (31.7%) | 2.77 | .429 | .067 |
| DTD | 194 (51.3%) | 127 (52.3%) | 0.02 | .898 | .005 |

Post-treatment total sample: males *n* = 378, females *n* = 243 (participants with unknown sex [*n* = 12] were excluded from all analyses).

No significant association between sex and post-treatment data availability was found for any outcome (all p > .13), with negligible effect sizes (all Cramér's V < .07).

*Note. BCIS = Beck Cognitive Insight Scale; CBQp-JTC = Cognitive Biases Questionnaire for Psychosis jumping-to-conclusions subscale; DTD = Draw-to-Decision (jumping to conclusions bias).*
